# Nodular panniculitis in a cat with high alpha tocopherol concentration in serum

**DOI:** 10.1002/vms3.286

**Published:** 2020-05-18

**Authors:** Martin Steffl, Nadine Nautscher, Alexander Kröpfl, Michael Granvogl

**Affiliations:** ^1^ Veterinary Practice of the University of Hohenheim Stuttgart Germany; ^2^ Institute of Food Chemistry, section Food Chemistry (170b) University of Hohenheim Stuttgart Germany; ^3^ Institute of Food Chemistry, section Food Chemistry and Analytical Chemistry (170a) University of Hohenheim Stuttgart Germany

**Keywords:** alpha tocopherol, cat, commercial food, panniculitis, serum, vitamin E

## Abstract

A 5‐year‐old male neutered domestic shorthair cat suffered from recurrent solitary nodules in different subcutaneous body regions. Nodules were surgically removed and each time histopathological diagnosis was fat necrosis and fibrosing to pyogranulomatous panniculitis. After the second surgery the alpha (α)‐tocopherol concentration in serum of the cat was examined and the result (21 mg/L) exceeded the upper limit of the reference interval (3–11 mg/L). Vitamin E amount in diet fed solely in the past was checked as studies have shown that vitamin E amounts in food significantly influence vitamin E concentrations in serum. For comparative purposes, α‐tocopherol concentrations were determined in sera of healthy control cats. Additionally, vitamin E amount in wet food from different manufacturers was analysed using gas chromatography coupled to mass spectrometry (GC‐MS). The results showed that the diet did not have higher vitamin E amounts compared to other diets. All control cats had similar high serum α‐tocopherol concentrations. We conclude that panniculitis can occur despite high serum α‐tocopherol concentrations in cats. Further studies are needed to redefine reference values of α‐tocopherol in serum of cats.

## Graphical abstract

To the best of our knowledge, this is the first case report of panniculitis in a cat despite its high serum alpha (α)‐tocopherol concentration. Our findings suggest that high serum α‐tocopherol concentrations may not always be protective against pathological disorders including panniculitis.

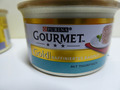

## 1 INTRODUCTION

Necrosis and inflammation of adipose tissue (panniculitis, steatitis, pansteatitis) in cats have been shown previously to result from an excessive dietary intake of unstabilized polyunsaturated fatty acids found in fish and fish by‐products (Gaskell, Leedale, & Douglas, 1975; Niza, Vilela, & Ferreira, 2003). Most commercial cat foods contain sufficient antioxidant vitamin E to stabilize the polyunsaturated fatty acids if they are formulated to meet nutrient requirements or industry recommended nutrient profiles. Interestingly, there were reports of pansteatitis in cats fed fish‐based commercial food that was not sufficiently supplemented with vitamin E (Cropper, 1980). It was suggested that low levels of vitamin E in the diet might be the result of poor control during manufacture or storage (Cropper, 1980).

Apart from vitamin E deficiency, panniculitis may have several possible aetiologies (Patterson, 2004). It has been associated with infectious agents (bacteria, fungi), pancreatic disease, vasculitis, immunological conditions, adverse drug reactions and physiochemical factors (trauma, foreign body, injections). However, panniculitis is often sterile and idiopathic (Patterson, 2004).

In this case report, we describe a 5‐year‐old male neutered cat suffering from recurrent nodular panniculitis. The lesions consisted of singular firm nodules existing at different subcutaneous body regions. As vitamin E deficiency is a common cause of fat necrosis/panniculitis, the alpha (α)‐tocopherol concentration in the cat serum was measured. It was shown that α‐tocopherol is the most active form of vitamin E and the second most common form of vitamin E in the diet (Rigotti, 2007). Surprisingly, the α‐tocopherol concentration was above the upper limit of reference interval (RI). Therefore, low serum α‐tocopherol concentration had been ruled out as a potential cause of panniculitis in this cat. The exact aetiology of panniculitis in this cat remains unclear, but possible causes and underlying mechanisms are discussed.

## 2 CASE DESCRIPTION

A male neutered domestic shorthair cat was presented for the first time with dermatological lesions at the age of 3 years. A nodule of pinhead size was palpated in subcutaneous tissue on the right chest. Clinical examination was unremarkable and complete blood chemical (VetScan2, Abaxis) and haematological (Scil Vet abc Plus) analyses were performed 3 weeks prior. Blood chemical parameters were within the RI, though leucocytosis of 23.9 K/µl (RI: 5.0–11.0 K/µl) was noted on haematology. Additionally, the feline leukaemia virus antigen test (enzyme‐linked immunoassay, Biocontrol) was negative. Specific antibodies against feline Coronavirus (1:1,600) measured by immunofluorescence assay (Biocontrol) and *Toxoplasma gondii* (*T. gondii* IgG: 1:1,024; IgM < 1:16) measured by indirect fluorescent antibody test (Biocontrol) were detected. Acute *T. gondii* infection was not considered due to the absence of clinical signs and specific IgM antibodies, respectively.

Three months later, the lesion reached a length of approximately (approx.) 2.5 centimetres (cm) and excisional surgery was performed under general anaesthesia. The removed tissue was submitted to a board‐certified pathologist for histopathological examination. Diagnosis was fat necrosis and fibrosing to pyogranulomatous panniculitis. Immunohistochemistry revealed no evidence for Coronavirus‐positive macrophages within the lesion. Therefore, feline infectious peritonitis (FIP) was excluded.

One year and seven months after surgery, the owner of the cat discovered a new firm nodule of approx. 1 × 1 cm in the region of the left abdomen. Cytological sampling via fine‐needle aspiration with a 22 G needle revealed inflammatory cells, which consisted mostly of neutrophils and macrophages, and reactive spindle cells with middle‐sized vacuoles indicating inflammation of adipose tissue. Six months later, the owner gave permission to perform excisional surgery again. The nodular structure was removed and submitted to histopathology. As expected, the diagnosis was fat necrosis and granulomatous to fibrosing panniculitis. As vitamin E deficiency is a common cause of fat necrosis/panniculitis, the serum α‐tocopherol concentration of the cat was determined. Additionally, blood was taken from the cat's brother living in the same household and showing no dermatological signs. Serum α‐tocopherol concentrations were measured using high‐performance liquid chromatography (HPLC, Biocontrol) and results showed high amounts of α‐tocopherol in serum of both cats (21 mg/L and 25 mg/L, respectively (RI: 3.0–11 mg/L)). According to owner details, both cats were fed almost exclusively with canned food (Purina Gourmet Gold) and preferred the tuna‐based flavour (table 1). The owner was asked to provide stored food for further chemical analyses. Additionally, vitamin E amount was determined in two purchased commercial fish‐based foods (Coshida, Almo Nature) for comparative purposes.

**TABLE 1:** Determination of vitamin E amount in fat (µg/mg) in different commercial cat foods. Values are converted in IU/100 g of food (in parentheses)^c^

Diet	Vitamin E (µg/mg)
Purina Gourmet Gold, refined ragout with tuna, 85 g, exp. day^a^ 12/2020; Analytical components: moisture 74%, protein 17%, fat 4%, ash 2%, fibre 1.1%; Additives: IU/kg: Vit. A (880), Vit. D3 (135)^b^	3.10 (18.48)
Purina Gourmet Gold, tender chunks veal & vegetables, 85 g, exp. day^a^ 05/2020; Analytical components: moisture 82%, protein 7%, fat 3.2%, ash 2.4%, fibre 0.1%; Additives: IU/kg: Vit. A (615), Vit. D3 (95)^b^	1.54 (7.34)
Almo Nature Daily, Adult cat with chicken and salmon, 70 g, exp. day^a^ 06/2020; Analytical components: moisture 82%, protein 9%, fat 5%, ash 3%, fibre 0.8%; Additives: IU/kg: Vit. D3 (238), mg/kg: Vit. E (23), Vit. B (1)^b^	5.18 (38.59)
Lidl Coshida, pâté with salmon, 100 g, exp. day^a^ 07/2020; Analytical components: moisture 81%, protein 10.5%, fat 4.5%, ash 1.8%, fibre 0.3%; Additives: IU/kg: Vit. D3 (200), mg/kg: Vit. E (100)^b^	31.56 (211.61)

^a^ expiration day

^b^ Vit = vitamin

^c^ NRC (1987): maximum tolerable level: 1,000 to 2,000 IU/kg diet

### 2.1 Saponification of the dietary lipid extract

Soxhlet extraction was followed by saponification of the lipid extract according to Grebenstein and Frank (2012) with slight modifications. In detail, lipids obtained from the cat food were transferred into a brown glass screw cap derivatization tube. Then, pyrogallol (2 ml; 60 g/L, w/v, dissolved in ethanol), water (900 µl) and saturated potassium hydroxide solution (300 µl) were added to the tube. Additionally, the tube was flushed with nitrogen to reduce oxidative stress. Then, the solution was saponified for 1 hr at 70°C in a sand bath and shaken vigorously every 5 min. The tube was cooled on ice and BHT (25 µl; 1 mg/ml, w/v, dissolved in ethanol), water (1 ml) and glacial acetic acid (300 µl) were added. The unsaponifiable matter containing the vitamin E was extracted with *n*‐hexane (2 ml). For this purpose, the tube including the saponification solution and *n*‐hexane was shaken 1 min by hand. After phase separation, an aliquot (500 µl) of the upper organic phase was transferred into a 1.5‐mL screw cap vial and diluted with *n*‐hexane (1/10, v/v), if necessary.

### 2.2 Gas chromatography‐mass spectrometry (GC‐MS)

The abovementioned aliquot of the unsaponifiable matter was trimethylsilylated according to Hammann, Englert, Müller, and Vetter (2015). The solvent was removed by means of an evaporator under a gentle stream of nitrogen at 40°C. Then, pyridine (25 µl) and BSTFA/TMCS (50 µl) were added, and the vial was heated to 60°C for 30 min. The solvent was again removed using nitrogen and taken up in a 5α‐cholestane solution (1 ml; 4 µg/ml, w/v, dissolved in *n*‐hexane). GC‐MS analysis was performed via a 6890/5973N GC‐MS system (Agilent) equipped with a HP5‐MS column (30 m × 0.25 mm i.d., 0.25 µm film thickness; Agilent) as described by Hammann and Vetter (2016). The following temperature programme was used: 60°C, raised at 20°C/min to 255°C, at 1.5°C/min to 283°C and finally at 15°C/min to 300°C (held for 5 min), leading to a total run time of 35.6 min. Mass spectra were collected in selected ion monitoring (SIM) mode with characteristic *m/z* values of all possible vitamin E forms, also including two ions for the internal standard 5α‐cholestane. α‐tocopherol was used as external standard. The calibration curve was assembled with four values reaching from 0.79 to 11.3 µg/ml.

Descriptions of the foods and results of the GC‐MS analysis are presented in Table 1. In summary, vitamin E amounts in Purina Gourmet Gold diets were lower than in control diets of Almo Nature and Coshida. The results of food chemical analyses revealed no explanation for the high amounts of α‐tocopherol in the blood of both cats. For this reason, serum α‐tocopherol concentrations of three healthy cats presented at Veterinary Practice for routine check‐ups were analysed to represent serum α‐tocopherol status in the local cat population. Serum α‐tocopherol concentrations of healthy control cats ranged from 21 to 29 mg/L (RI: 3.0–11 mg/L) (table 2). At this point, low serum α‐tocopherol concentration had been ruled out as a potential cause of panniculitis in this cat, as the serum levels found were similar to his unaffected brother in the same household as well as three other cats obtained from the local cat population. However, panniculitis has been associated with numerous other causes, including infectious organisms, immunological conditions and physiochemical factors (Patterson, 2004). The pathologist interpreted the histopathological findings in this cat as the consequences of injuries. Therefore, solitary lesions may be cured by excision and further therapeutic measures are not necessary. The affected cat did not develop new lesions 1 year after the last (second) surgery until preparing the manuscript.

**TABLE 2:** Determination of serum α‐tocopherol concentration (reference interval: 3.0–11 mg/L, Baker et al., 1986) in the affected cat, the affected cat's brother and healthy control cats fed different diets according to owner statements

Cat, sex, age	Main food	α‐tocopherol (mg/L)
Domestic shorthair, male neutered, 5 year (affected)	Purina Gourmet Gold	21
Domestic shorthair, male neutered, 5 year (brother)	Purina Gourmet Gold	25
Maine Coon, female neutered, 4 year 3 months	Royal Canin Maine Coon	29
Maine Coon, male neutered, 9 year 9 months	Bozita, Purina One Sterilcat	21
Domestic shorthair, female neutered, 12 years	Royal Canin Urinary	23

## 3 DISCUSSION

Vitamin E deficiency was previously documented as a cause of fat necrosis/panniculitis in dogs and cats fed fish‐based diets (Cropper, 1980; Gaskell et al., 1975). However, the appearance of panniculitis due to vitamin E deficiency decreased because commercial foods are usually supplemented with vitamin E. Surprisingly, nodular panniculitis in the 5‐year‐old cat was found despite high, not low serum α‐tocopherol concentration. Additionally, cat's brother also had high serum α‐tocopherol concentration. We expected to find a high amount of dietary vitamin E in the cat's food because it was shown previously that increasing levels of dietary vitamin E in dog and cat foods caused significant increases in serum vitamin E levels (Jewell, Toll, Wedekind, & Zicker, 2000; Jewell, Yu, & Joshi, 2002). The results of food chemical analysis revealed no explanation for the high amount of α‐tocopherol in blood of both cats. Vitamin E amounts in Purina Gourmet Gold diets fed to both cats were lower than in control diets of Almo Nature and Coshida (Table 1). The current minimum recommendation for vitamin E in adult cats is 3.80 IU per 100 g dry matter (DM) based on maintenance energy requirement (MER) of 100 kcal/kg^0.67^ (FEDIAF, 2018). The National Research Council (NRC, 1987) suggested a maximum tolerable limit of 1,000 to 2,000 IU/kg diet. Thereby, it is important to know that no maximum levels are listed for cats. Thus, all four foods analysed represent a large range of low vitamin E foods and high vitamin E foods as similarly described by Jewell et al. (2002).

Sex and breed have no significant effect on concentration of plasma α‐tocopherol, but age‐dependent influence was noted previously (Fox et al., 1993; Fytianou, Koutinas, Saridomichelakis, & Koutinas, 2006). In healthy cats that consumed commercial foods, the plasma α‐tocopherol concentration has been shown to increase with age (Fox et al., 1993; Fytianou et al., 2006). However, these changes were not statistically significant in adult cats (Fox et al., 1993) and the mean plasma α‐tocopherol concentration in 4‐ to 6‐year‐old cats was dramatically lower (13.95 ± 0.826 mg/L, Fox et al., 1993) than described herein.

In addition to the affected cat and his brother, serum α‐tocopherol concentrations of three healthy cats presented at Veterinary Practice for routine check‐ups were also analysed to represent serum α‐tocopherol status in the local cat population. The results (Table 2) showed that serum α‐tocopherol concentrations of the healthy control cats ranged from 21 to 29 mg/L (RI: 3.0–11 mg/L). The cat with panniculitis and cat's brother had similar high values (Table 2). It appears that the reference interval established for more than 30 years (Baker et al., 1986) should be re‐evaluated. Baker et al. (1986) used plasma for vitamin E (not α‐tocopherol) determination and their chemical methods derived from the year 1968. Interestingly, Jewell et al. (2002) showed that the serum vitamin E amounts in cats fed diets rich in vitamin E (709 IU/kg of food) were comparable to the serum vitamin E amounts found in our case study. It appears that vitamin E and/or other dietary factors in the diet may influence serum vitamin E concentration (Hendriks et al., 2002). For example it has been shown that selenium has a sparing effect on α‐tocopherol concentration. Clinical signs of vitamin E deficiency in growing dogs fed a low vitamin E diet were prevented by supplementation of selenium at 0.05 ppm (Van Vleet, 1975).

Vitamin E deficiency had been ruled out as a potential cause of panniculitis in this cat. However, panniculitis has been associated with numerous other causes, including infectious organisms, immunological conditions and physiochemical factors (Patterson, 2004). The histopathological examination revealed no evidence for infectious agents including bacteria and fungi, albeit special stains were not performed. Pyogranulomatous vasculitis induced by feline Coronavirus (Declercq, Bosschere, Schwarzkopf, & Declercq, 2008) was also excluded by immunohistochemistry. Moreover, there were no clinical and laboratory signs (e.g. elevation of serum amylase) for pancreatic disease. Panniculitis secondary to pancreatitis is regarded to be rare in the cat (Kunder & Foster, 2016). Drugs were not administered. The board‐certified pathologist interpreted the histopathological findings in this cat as the consequences of injuries. In fact, traumatic events often occur in out‐door cats and include cat fights, penetrating foreign bodies or traffic collisions. However, skin injuries or haematomas were not found in this cat. Interestingly, the second nodular structure was presented 3 months after routine vaccination. Therefore, it is possible that panniculitis in this cat was induced by injection as a traumatic event.

## 4 CONCLUSION

Clinicians should interpret reference values of α‐tocopherol in serum of cats with the understanding that there is no recognized hypervitaminosis E or toxicity associated with dietary α‐tocopherol in cats. Studies have shown that vitamin E amounts in food significantly influence vitamin E concentrations in serum. High α‐tocopherol concentrations in serum are not necessarily protective against pathological disorders including panniculitis. However, further studies are needed to re‐evaluate the reference interval of α‐tocopherol in serum of cats.

## CONFLICT OF INTEREST

The authors declared no potential conflicts of interest with respect to the research, authorship, and/or publication of this article.

## AUTHOR CONTRIBUTION

**Martin Steffl:** Conceptualization; Investigation; Resources; Writing‐original draft. **Nadine Nautscher:** Investigation; Writing‐review & editing. **Alexander Kröpfl:** Investigation; Validation. **Michael Granvogl:** Investigation; Validation; Writing‐review & editing.

### Peer Review

The peer review history for this article is available at https://publons.com/publon/10.1002/vms3.286.
